# Cancer-Associated Thrombosis: An Overview of Mechanisms, Risk Factors, and Treatment

**DOI:** 10.3390/cancers10100380

**Published:** 2018-10-11

**Authors:** Norbaini Binti Abdol Razak, Gabrielle Jones, Mayank Bhandari, Michael C. Berndt, Pat Metharom

**Affiliations:** 1 School of Pharmacy and Biomedical Sciences, Curtin University, Perth 6100, Australia; n.abdolrazak@postgrad.curtin.edu.au (N.B.A.R.); gabrielle.jones@postgrad.curtin.edu.au (G.J.); 2 Curtin Health Innovation Research Institute, Curtin University, Perth 6100, Australia; 3 Fiona Stanley Hospital, Perth 6150, Australia; Mayank.Bhandari@health.wa.gov.au; 4 School of Medicine, Curtin University, Perth 6100, Australia; m.berndt@curtin.edu.au

**Keywords:** venous thromboembolism, thrombosis, cancer

## Abstract

Cancer-associated thrombosis is a major cause of mortality in cancer patients, the most common type being venous thromboembolism (VTE). Several risk factors for developing VTE also coexist with cancer patients, such as chemotherapy and immobilisation, contributing to the increased risk cancer patients have of developing VTE compared with non-cancer patients. Cancer cells are capable of activating the coagulation cascade and other prothrombotic properties of host cells, and many anticancer treatments themselves are being described as additional mechanisms for promoting VTE. This review will give an overview of the main thrombotic complications in cancer patients and outline the risk factors for cancer patients developing cancer-associated thrombosis, focusing on VTE as it is the most common complication observed in cancer patients. The multiple mechanisms involved in cancer-associated thrombosis, including the role of anticancer drugs, and a brief outline of the current treatment for cancer-associated thrombosis will also be discussed.

## 1. Introduction

Armand Trousseau first reported on the relationship between thrombosis and cancer in 1865. Since then, numerous studies have established that thrombosis is a common complication for cancer patients, contributing to the second-leading cause of mortality in cancer patients [1,2]. Thrombotic complications in cancer can vary from arterial or venous thromboembolism to disseminated intravascular coagulation [3,4]. Despite the well-known association between cancer and thromboembolic disease, the mechanisms that promote thromboembolic events in cancer patients are not clear and appear to be multifaceted [5]. Cancer patients are generally in a hypercoagulable or prothrombotic state, as they usually present with abnormalities in each component of Virchow’s triad, thus contributing to thrombosis. The three components are a stasis of blood flow, endothelial injury, and hypercoagulability, the latter including abnormalities in the coagulation and fibrinolytic pathway and platelet activation. The specific mechanisms leading to abnormalities in Virchow’s triad in cancer patients, particularly the effect on the host haemostatic system to promote the prothrombotic state, are not well understood and may be tumour specific as different cancer types have varying risk rates for cancer-associated thrombosis. This review will give an overview of the main thrombotic and bleeding disorders in cancer (arterial and venous thrombosis and chronic disseminated intravascular coagulation), the risk factors for developing cancer-associated thrombosis, and the multiple mechanisms (direct and indirect) thought to promote cancer-associated thrombosis. A brief outline of the current treatment of cancer-associated thrombosis will also be discussed.

## 2. Types of Cancer-Associated Thrombosis

### 2.1. Venous Thromboembolism

Venous thromboembolism (VTE) comprises deep vein thrombosis (DVT) and pulmonary embolism (PE). The development of VTE is often initiated in the valve sinus where a number of features surrounding these valves make the site prone to thrombosis. These include abnormal and reduced blood flow, reduced shear stress, and hypoxia leading to an intact but dysfunctional endothelium [6]. In addition, platelets and leukocytes tend to become trapped in valve pockets [7]. In cancer patients, tumours can compress veins, resulting in venous stasis, thus encouraging thrombosis. VTE contributes significantly to morbidity and mortality of cancer patients, with a fatal PE being 3 times more common in cancer patients compared to non-cancer patients [8,9]. Cancer patients have a 5- to 7-fold increased risk of developing VTE [10,11] and those who develop VTE at diagnosis of cancer or within the year tend to have a significantly worse prognosis compared with cancer patients without VTE [12]. A diagnosis of VTE is a serious complication of cancer that adversely affects a patient’s quality of life and reduces overall survival rates [13,14]. It is estimated that approximately 4–20% of cancer patients will experience VTE at some stage, the rate being the highest in the initial period following diagnosis. Annually, 0.5% of cancer patients will experience thrombosis compared with a 0.1% incidence rate in the general population [15].

### 2.2. Arterial Thrombosis

Although there are fewer data available on arterial thrombosis in cancer compared with on VTE, it is nonetheless observed in cancer. There have been multiple case reports suggesting acute arterial thrombosis in the setting of a new malignancy [16]. Navi et al. recently investigated the association between cancer patients and risk of arterial thrombosis in a large retrospective matched-cohort study. The incidence rate of arterial thrombosis at 6 months was 4.7% in cancer patients compared with 2.2% in the matched controls [17].

The pathogenesis of arterial thrombosis differs substantially from venous thrombosis as it typically occurs with endothelial damage. An atherosclerotic plaque is prone to thrombosis when it presents as a lipid-rich core with a thin fibrous cap. A thrombus can form over a ruptured plaque or an intact plaque that has superficial endothelial erosion [18]. Platelet activation that is persistent at the site of rupture promotes thrombosis by the exposure of procoagulant molecules within the plaque core. In contrast to the low venous shear rates in VTE development, the high shear rates in stenosed arteries contribute to a thrombus that is predominantly composed of platelets, as they are the only blood component capable of adhering at high shear. The high shear rates can also activate platelets, thus further promoting thrombosis [19]. The resulting intraluminal thrombosis often manifests as myocardial infarction or stroke. Furthermore, tissue factor found in atherosclerotic plaques appears to play an important role in the initial development of thrombosis following plaque rupture [18]. It should be noted, however, that arterial thrombosis in cancer can occur in the absence of an atherosclerotic plaque such as that observed in cardiovascular patients, where systemic hypercoagulation is induced by several secreted factors from cancer cells, such as thrombin and vascular endothelial growth factor (VEGF), thereby promoting platelet activation and coagulation [20].

Many chemotherapeutic agents are known to be prothrombotic and there are multiple case reports documenting an association between chemotherapy and arterial thrombosis. Platinum-based agents (cisplatin), vascular endothelial growth factor (VEGF) inhibitors (bevacizumab), and VEGF tyrosine kinase receptor inhibitors (sorafenib/sunitinib/pazopanib) have been associated with increased rates of arterial thrombosis [20]. Other major risk factors for arterial thrombosis include vessel damage which results from hypertension, atherosclerosis, or vascular anomalies. These factors contribute to arterial thrombosis by inducing turbulence and altering blood flow, hence allowing for platelet adhesion which plays an important role in pathogenesis [18]. Furthermore, a recent meta-analysis has shown that the major risk factors for arterial thrombosis are significantly associated with VTE [21], suggesting that the two thrombotic disorders are simultaneously activated by biological stimuli.

### 2.3. Chronic Disseminated Intravascular Coagulation

Thrombotic complications observed in cancer are not limited to VTE or arterial thrombosis, with other more severe manifestations of the procoagulant state such as disseminated intravascular coagulation (DIC) and thrombotic microangiopathy (TMA) [12,22,23]. DIC is a severe yet rare complication of cancer that manifests itself as a consumptive coagulopathy resulting in microvascular thrombosis with tendency for severe bleeding, thrombocytopaenia, and organ failure [24,25]. Bleeding is thought to be due to hyperfibrinolysis that dominates microvascular thrombosis [26]. The incidence of DIC in solid tumours reported in a clinical study was 7% [27], with other reports indicating high incidence of up to 85% in acute promyelocytic leukaemia [28] Treating cancer patients with acute DIC is often very difficult with the majority of patients dying within 1–4 weeks [24]. DIC and thrombogenesis in cancer patients tends to involve defects in the three constituents of normal host defense against thrombosis: (1) blood flow leading to stasis, (2) balance of the procoagulant and anticoagulant proteins within the blood, resulting in the activation of circulating procoagulant proteins, and (3) vessel wall activation, resulting in an increase in its procoagulant contribution [12]. The clinical presentation of DIC in cancer patients is often less severe and has a more delayed onset, but following the clinical presentation, DIC progresses in a gradual yet chronic manner whereby systemic activation of coagulation occurs [22]. Eventually, this process can result in the exhaustion of coagulation factors and platelets, and bleeding may occur as the first clinical symptom to indicate DIC [22].

DIC is often difficult to distinguish from thrombotic microangiopathy (TMA), which manifests as thrombotic purpura and haemolytic uremic syndrome [23]. TMA shares the same clinical consequences as DIC and, thus, also leads to microvascular thrombosis with increased tendency for bleeding and organ failure. However, although DIC and TMA are similar and associated with each other, it is important that they be differentially diagnosed as treatments for TMA and DIC are different [25]. DIC is brought about by the marked activation and consumption of the coagulation system (triggered by substances like tissue factor, inflammatory cytokines, and activation leukocytes) which subsequently activates secondary fibrinolysis [25]. However, TMA onset is initiated by the marked activated and consumption of platelets in response to numerous factors which in turn lead to activation and subsequent injury to vascular endothelial cells [25].

## 3. Risk Factors for Cancer-Associated Thrombosis

Cancer is a well-known and established risk factor for thromboembolic events; however, there are several other risk factors known to increase a cancer patient’s risk of developing thrombosis. In cancer patients, arterial and venous thrombosis are considered clinical manifestations of a multifactorial systemic disease and result from a number of risk factors which can be divided into the individual patient and cancer-associated risks [29]. Such risk factors include age, ethnicity, immobility, cancer type, and chemotherapy, will be discussed briefly below. Due to VTE being much more frequently clinically observed in cancer patients relative to arterial thrombosis, this section will concentrate on risk factors for VTE.

### 3.1. Individual Patient Risk Factors

#### 3.1.1. Age

In the general population, incidence rates for VTE increase exponentially with age [30,31]. A large prospective study found that individuals aged 85 years and older have an almost 10-fold higher incidence rate (6.96 per 1000 person-years) compared with those aged 45 to 54 years of age (0.72 per 1000 person-years) [31]. Likewise, increasing age is a risk factor for VTE in the cancer population. In retrospective cohort studies, cancer patients aged ≥65 years old have a greater likelihood of developing VTE compared with younger patients [32,33]. Similarly, in patients undergoing cancer surgery, the risk of VTE increased with age, compared with those aged less than 60 years (OR = 2.6, 95% CI: 1.2–5.7). In a retrospective cohort study undertaken by Vergati and colleagues that included cancer patients that were undergoing chemotherapy, the elderly patients (>70 years) had an almost 2-fold increased risk of developing VTE compared with the young to middle-aged patients (≤70 years), with incidence rates of 11% and 6%, respectively [34]. Increasing age, irrespective of cancer, is accompanied by factors that increase one’s risk of thrombosis, including decreased exercise, increased immobility, and systemic activation of coagulation (reviewed in [35]).

#### 3.1.2. Sex

Few studies have looked at the overall effect of sex in a cancer cohort and the risk of developing VTE. Retrospective studies show that females are at greater risk for VTE [32], while male patients are more likely to develop arterial thromboembolism [33]. In contrast, Chew et al. [36] found that sex did not predict thromboembolism in any of the cancers included in their study.

#### 3.1.3. Race

In a large retrospective study by Khorana et al. [32], the rates of VTE occurrence in cancer patients of different ethnicities were examined. A significant association with VTE was found in black patients, who had the highest rate of VTE (5.1%), followed by whites and Hispanic patients (4.0%). The lowest rates were observed in Asian/Pacific Islander patients (3.3%), consistent with Chew et al. [36]. Moreover, the rate of VTE in black patients increased at a greater rate (36.7%) than in other ethnicities (26.8%). In contrast, a large retrospective study found no significant differences in the incidence rates of PE and DVT between blacks and whites [37].

#### 3.1.4. Comorbidities

Multiple studies have identified an association between medical comorbidities and an increased risk of cancer-associated thrombosis. Comorbid conditions such as renal failure, respiratory disease, heart disease, obesity, and acute infection have been found to be associated with an increased risk of developing VTE in cancer patients, with infection identified as one of the most strongly associated risk factors for VTE [9,32].

#### 3.1.5. Immobility

Immobility plays a role in predisposing cancer patients to VTE. Mobility in cancer patients is clinically assessed by performance status, and higher rates of VTE were observed in cancer patients with poor performance status [38,39]. In another study, bed rest of greater than 3 days was associated with significantly higher rates of VTE [40]. It is believed that immobility increases the chance of VTE through stasis of the venous blood flow [41].

#### 3.1.6. Previous History of VTE

A previous episode of VTE is a major risk factor for developing VTE. Cancer patients with a history of VTE have a 6- to 7-fold increased risk of VTE recurrence when compared with cancer patients with no history of VTE [40,42].

### 3.2. Cancer-Associated Risk Factors

The rates of cancer-associated thrombosis (CAT) can be impacted by a number of cancer-related risk factors including cancer site, stage, histological subtype, and time since diagnosis. It is important to note that comparison of VTE rates amongst different patients in the literature is often difficult due to varying differences in study design, patient populations, methods for data collection, and follow-up periods [42].

#### 3.2.1. Site of Cancer

The primary site of the cancer is frequently identified as a risk factor for VTE, with cancers of the pancreas, uterus, lung, stomach, and kidney, and primary brain tumours associated with an increased risk of VTE [43]. A recent meta-analysis found that pancreatic cancer displayed the highest rate of VTE [44], while other studies suggest that the highest incidence rates occur in mucin-producing adenocarcinomas of the pancreas, lung, and gastrointestinal tract [45]. In contrast, the rates of increased VTE in cancer patients may reflect the frequency of cancer within the population as a whole, with a higher incidence of CAT observed in women with ovarian, breast, and lung cancer and higher incidence observed in men with prostate, colorectal, and lung cancer [45]. Irrespective of these differences, it is universally agreed upon that the incidence of VTE is not equivalent in different cancer types, which may suggest cancer-specific mechanisms playing a role in cancer-associated thrombosis.

#### 3.2.2. Stage of Cancer

Patients with advanced-stage cancer appear to be at a greater risk of developing VTE [9]. In a Danish population-based cohort study, the risk of cancer patients developing VTE increased with cancer stage, and the calculated adjusted relative risks for stage I, II, III, and IV cancer were 2.9, 2.9, 7.5, and 17.1, respectively [46]. Similarly, a study observing oncologic patients who underwent surgery reported that patients with advanced-stage cancer had a higher risk of developing VTE [40]. A population-based study also found that patients with distant metastases are at a greater risk of VTE compared with patients without distant metastases. This study reported an initial 4-fold increased risk in cancer patients without metastases compared with non-cancer patients, which increased to 58-fold in patients who had solid tumours with distant metastases [11]. More recently, data from the Vienna Cancer and Thrombosis Study comparing the effect of local, regional, and distant cancer stages on development of VTE found a significantly higher risk of VTE in patients with regional and distant disease, when compared with patients with local disease [47].

#### 3.2.3. Histology of Cancer

Histological subtypes of some types of cancer have been linked to an increased risk of VTE. For example, histological subtypes of lung and ovarian cancer show varying degrees of increased risk for VTE, while other histopathological subtypes of colon and breast cancer are nonpredictive for cancer-associated VTE incidence [14]. Studies have shown an increased risk of adenocarcinoma compared with squamous cell carcinoma in patients with non-small cell lung cancer [36,48]. Some studies have reported that the mucin-producing adenocarcinomas, e.g., pancreas, lung, and gastrointestinal tract, have the highest incidence of cancer-associated VTE [45]. On the other hand, there was no significant difference in the incidence of VTE in different histological subtypes of breast and colon cancer [49,50].

Ahlbrecht et al. published results from the Vienna Cancer and Thrombosis study and reported that tumour grade was associated with an increased risk of VTE in a cohort of patients’ variety of solid tumours [51]. Patients with high-grade tumours (G3 and 4) had 2-fold increased risk of developing VTE compared with those with low-grade tumours (G1 and 2). Thus, tumour grading may be another valuable histopathologic parameter to stratify patients into VTE risk groups.

#### 3.2.4. Time after Diagnosis

The immediate period following a cancer diagnosis is when the risk of developing VTE is highest [43]. This could be explained by the fact that many therapeutic interventions occurring during this period of time, such as chemotherapy, carry their own increased risk [43]. Numerous studies have demonstrated that the initial period following a cancer diagnosis is linked to a higher risk of VTE with the greatest risk during the first three to six months following diagnosis, while other research suggests that the risk of VTE is greatest within the first year following diagnosis [9,14,52].

### 3.3. Cancer-Treatment-Associated Risk Factors

#### 3.3.1. Surgery and Hospitalisation

The prothrombotic state of malignancy is often provoked by cancer therapies and treatments, including surgery. Historically, the observed incidence of VTE has been greatest in cancer patients admitted to the hospital, either for surgery or acute medical illness [53]. VTE is a common complication for those patients who undergo surgery, with cancer surgery increasing the risk of postoperative DVT 2-fold and that of fatal PE greater than 3-fold when compared with similar procedures carried out on non-cancer patients [40]. Over the years, the risk for thrombotic complications as a result of cancer surgery has been reduced due to improved surgical techniques, increased mobility following surgery, improvement of prophylaxis, and improvements in perioperative care [40]. Cancer-related surgery of the pelvis and abdomen is associated with a higher risk for VTE development in patients, while orthopaedic surgery of the lower limbs is also considered a high-risk surgery, particularly in Western populations [54,55].

#### 3.3.2. Chemotherapy

Chemotherapy is an important risk factor for VTE amongst cancer patients and may explain its increasing incidence over the past few decades [53]. Cancer patients have a 6- to 7-fold increased risk of developing chemotherapy-associated thrombosis [53]. A predictive model for chemotherapy-associated VTE was developed and ranks patients with stomach and pancreatic cancers as very high risk compared with other cancer types. These patients are assigned 2 points, and a score greater than 2 classifies a patient into the high-risk VTE group [56]. A study undertaken by Khorana et al. determined that VTE rates were significantly higher in cancer patients over the 12 months following the initiation of chemotherapy compared with in non-cancer patients [53]. In cohort studies, patients who were receiving or were recently exposed to chemotherapy were found to be at increased risk of developing thrombosis [46,57]. Many types of chemotherapy drugs used to treat cancer have been associated with an increased risk of thrombotic events. Cisplatin is a platinum-based chemotherapeutic drug widely used in several malignancies and is usually used in combination with other drugs. The first evidence of cisplatin inducing increased arterial vascular toxicity and thrombotic potential was in 1986 [58], not long after its FDA approval in 1976. Substantial risk of both venous and arterial thromboembolic events has been reported in patients after treatment with cisplatin-based chemotherapy [59,60]. Combination chemotherapy with cisplatin leads to an approximately 2-fold increase in thrombotic complications in gastroesophageal cancer patients compared with patients receiving combination therapy with another platinum-based drug, oxaliplatin [61]. Other immunosuppressive or cytotoxic chemotherapies, such as L-asparganase, thalidomide, lenalidomide, and tamoxifen, have also been reported to increase the risk of VTE [62].

#### 3.3.3. Angiogenesis Inhibitors

The importance of angiogenesis in tumour growth, invasion, and metastasis has led to the use of inhibitors that target blood vessel formation. Bevacizumab is a monoclonal antibody targeting circulating vascular endothelial growth factor (VEGF), which can be released from cancer cells and activates the endothelium. The effect of bevacizumab appears to have a greater impact on increasing the risk of arterial rather than venous thromboembolic events. While it is unclear if this drug increases the risk of VTE [63], several meta-analyses and large clinical trials have found bevacizumab to increase the risk of arterial thromboembolic events when compared with chemotherapy alone [64,65].

#### 3.3.4. Central Venous Catheters

Central venous catheters (CVC) are vital for a number of aspects of cancer therapy, including delivery of intravenous drugs and collecting blood samples [14,66]. Their use, however, can result in the formation of catheter-related thrombosis, which is a serious complication that can interrupt the infusion of chemotherapy treatment, blood products, or intravenous medications, as well as cause serious morbidity including PE and postphlebitic syndrome [66,67]. It is estimated that the incidence of catheter thrombosis is between 5 and 30%, but it is thought to be underestimated because clinical signs of catheter thrombosis appear to be vague and nonspecific [66]. Indwelling CVC have been linked to an increased risk for developing VTE, and it is estimated that the rate of symptomatic catheter-related DVT is between 0.3 and 28%. However, this number dramatically increases to approximately 27 to 66% using venography [14].

## 4. Mechanisms of Cancer-Associated Thrombosis

**Figure 1:**
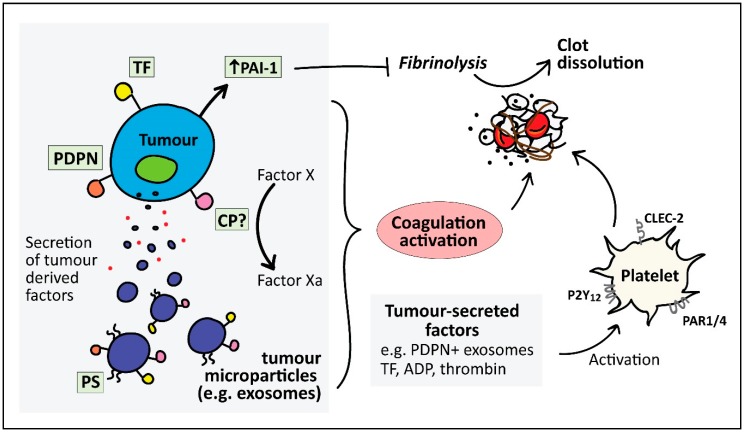
Direct mechanisms involved in cancer-associated thrombosis. Direct activation of coagulation and platelets can occur through several factors expressed on or released from cancer cells. These include the expression of tissue factor (TF), the key initiator of the coagulation cascade, which can also be released by TF-positive microparticles. Podoplanin (PDPN) expression can directly cause platelet activation and aggregation via the C-type lectin-like receptor 2 (CLEC-2) receptor on platelets. Plasminogen activation inhibitor-1 (PAI-1), a key inhibitor of fibrinolysis, is highly expressed in cancer cells. Cancer cells also secrete platelet agonists such as ADP and thrombin, thus further promoting platelet activation through P2Y12 and protease-activated receptors 1 and 4 (PAR1/4), respectively. Phosphatidyl serine (PS) expressed on tumour microparticles may also promote coagulation as PS serves as a surface for formation of coagulation complexes. Cancer procoagulant (CP) has been shown to directly activate coagulation by activating Factor X.

**Figure 2:**
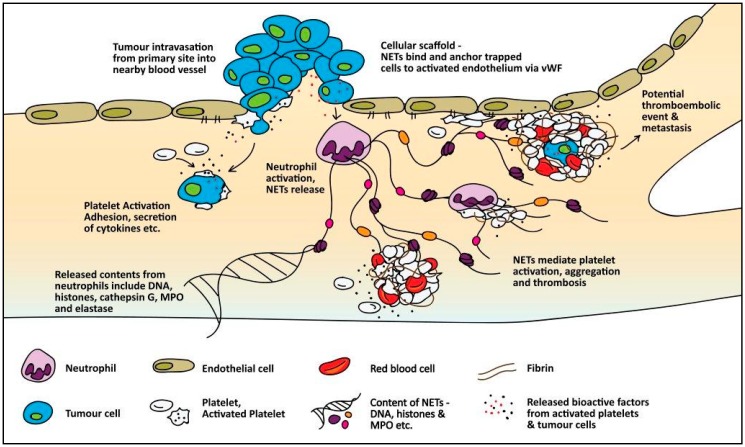
Indirect mechanisms promoting thrombosis in cancer. Tumours can be highly metastatic, resulting in cancer cell dissemination and intravasation into nearby blood vessels. Inflammatory cytokine secretion from tumour cells can cause activation of platelets and promote a procoagulant phenotype in endothelial cells. Cancer-derived factors also stimulate neutrophils to release neutrophil extracellular traps (NETs). NETs serve as a scaffold that can physically entrap platelets, or activate platelets through NET-associated histones, ultimately leading to profound platelet activation, fibrin deposition, and entrapment of red blood cells, exacerbating clot formation.

The molecular mechanisms leading to the predisposition of cancer patients to thromboembolic events are poorly understood. However, several mechanisms that can promote a hypercoagulable state are seen in cancer patients, thereby increasing the risk of thromboembolic events. This section will focus on both direct (Figure 1) and indirect (Figure 2) mechanisms where a number of studies which the readers are referred to are pancreatic or stomach cancer studies.

### 4.1. Direct Mechanisms for Cancer-Associated Thrombosis

#### 4.1.1. Tissue Factor

Tissue factor (TF) is the best characterised tumour-derived procoagulant protein. It is a 47 kDa transmembrane protein that primarily initiates the extrinsic pathway of the coagulation cascade, resulting in thrombin generation which activates platelets and initiates blood clotting [68]. TF is abundantly expressed on subendothelial cells such as fibroblasts, pericytes, and vascular smooth muscle cells, and triggers haemostasis upon vascular injury. However, TF is not expressed on normal quiescent endothelium. In contrast, malignant tissue involving endothelial and tumour cells constitutively expresses TF. Despite many observations of TF expression in several types of cancers, the association of tumour TF expression with risk of VTE has only been observed in pancreatic and ovarian cancer [69,70]. Furthermore, TF expression has been shown to increase with advanced stages of pancreatic cancer and poorer prognosis [71,72]. Apart from TF expression in pancreatic tumours, TF is also present on the surface of microvesicles that are released by pancreatic tumours, which was also found to be associated with increased VTE in pancreatic cancer patients [73].

#### 4.1.2. Microparticles (MP)

MP released from cancer cells can serve as direct and indirect contributors to the prothrombotic mechanism in cancer. MP are small (0.1–1 µm diameter) membrane vesicles that are released from apoptotic or activated normal cells, or resting malignant cells. Early studies showed that breast and hepatocarcinoma cell lines showed procoagulant activity both in vivo and in vitro, which was associated with tumour-shed vesicles [74]. More recently, circulating MP in several cancer types have been shown to accelerate thrombus formation in vivo [75]. The procoagulant activity of MP has been attributed to the surface expression of active TF [75,76,77], as well as the presence of phosphatidylserine, which provides a negatively charged surface that supports the assembly of coagulation complexes [78]. Recently, Stark et al. reported phosphatidylethanolamine externalisation from pancreatic cancer MP as an important player in cancer-associated DVT [79]. In addition, Geddings and colleagues showed that TF-positive MP enhanced platelet activation and increased thrombosis in mice [76]. The association of TF-positive MP and VTE incidence has only been observed in pancreatic cancer patients [80].

#### 4.1.3. Podoplanin

Cancer-associated fibroblasts express podoplanin [81,82], a protein known to cause activation and aggregation of platelets, referred to as tumour-cell-induced platelet activation, through the C-type lectin receptor 2 (CLEC-2) [83]. Podoplanin expression has been reported in several pancreatic cancer cell lines, HPAF-II, HPAC and PL45 [84], and BxPC-3 (unpublished laboratory data). In an inferior vena cava (IVC) stenosis model of DVT, CLEC-2 depletion in platelets leads to reduced venous thrombosis, which was restored after transfusion of wild-type platelets. Increased podoplanin levels in the IVC wall after stenosis correlated with the degree of thrombosis [85]. However, the association of tumour-associated podoplanin and VTE in cancer patients has not been established apart from in patients with brain cancer [86]. It has been suggested that cancer cells expel podoplanin into the bloodstream in order to have an effect on thrombosis at distant sites [87]. Supporting this, tumour-derived MPs that bear podoplanin have been detected in the blood of patients with pancreatic and colorectal cancer [88].

#### 4.1.4. Plasminogen Activator Inhibitor-1 (PAI-1)

PAI-1 is a key inhibitor of fibrinolysis which has been shown to be highly expressed in pancreatic cancer cells [89]. Increased PAI-1 in plasma results in reduced fibrinolytic activity, thus increasing the risk of thrombosis [90]. A study conducted in 1992 found excess PAI-1 in pancreatic cancer patients which correlated with thromboembolic developments [91]. However, the role of the fibrinolytic system and PAI-1 in cancer-associated thrombosis has not been well studied. A study investigating the role of PAI-1 in a murine xenograft A549 cell tumour model found significantly increased thrombi and shortened occlusion times when treated with an anti-VEGF drug, bevacizumab, which also increased PAI-1 levels in the tumour and plasma [92]. Interestingly, the increased thrombotic effect of bevacizumab was significantly reduced by a PAI-1 inhibitor, suggesting a role of PAI-1 in cancer-associated thrombosis. However, further studies are required to elucidate its role and mechanism.

#### 4.1.5. Cancer Procoagulant (CP)

CP is a cysteine protease that was firstly isolated from rabbit malignant tissue and reported to induce direct coagulation activation by directly activating factor X, without a requirement for coagulation factor VII [93]. It was later isolated from human carcinomas carrying procoagulant activity [94]; however, a study in breast cancer patients found no association of CP with procoagulant markers [95]. The purity of the CP preparations used in earlier studies that established CP as a procoagulant protein was later questioned as potential contamination of TF/factor VIIa complex [96]. Hence, Raasi et al. conducted a study to examine the presence of TF associated with CP by sequencing proteins that reacted with anti-TF monoclonal antibodies. Although there were cross-reactive proteins, none resembled the molecular weight or sequence of TF [97]. The role of CP as a procoagulant in cancer has gone largely unstudied and more studies would be required to confirm the role of CP as a protein that activates coagulation and its association with cancer-associated thrombosis.

#### 4.1.6. Tumour-Derived Platelet Agonists

Adenosine diphosphate (ADP) and thrombin are well-known platelet aggregation agonists. Cancer cells are known to secrete ADP which causes platelet activation and aggregation via the P2Y1 and P2Y12 receptors [98]. Thrombin is also generated by pancreatic tumours [99] and has been found to be increased in plasma of patients with pancreatic cancer [100], thus implicating a role of these tumour-derived products in platelet activation and coagulation in pancreatic cancer.

### 4.2. Indirect Mechanisms of Cancer-Associated Thrombosis

Both cancer-derived factors and associated mechanisms that can activate or facilitate interactions with host cells can subsequently promote thromboembolic events. The host cells that have main roles in promoting thrombosis in cancer include platelets, leukocytes, and endothelial cells. A summary of indirect mechanisms that can promote thrombosis in cancer is depicted in Figure 2.

#### 4.2.1. Microparticles

As described above, TF-positive MP observed in cancer patients are derived from cancer cells. In addition, they can also be released from activated endothelial cells and monocytes in response to cancer. Inflammatory cytokine release from cancer cells can cause activation of endothelial cells and monocytes and stimulate the release of TF-positive MP; however, the relative contribution of tumour cells and host cells to the total amount of TF-positive MP observed in cancer patients is unknown.

#### 4.2.2. Inflammatory Cytokines

Tumour cells synthesise and secrete various inflammatory cytokines which are generally thrombogenic, capable of promoting a procoagulant phenotype in host endothelial cells [101]. In addition, the tumour presence causes a reactive response in host inflammatory tissues leading to excess cytokine release. The most well-defined proinflammatory cytokines that have been shown to exert prothrombotic effects are tumour necrosis factor alpha (TNF-α) and interleukin-1β (IL-1β). TNF-α and IL-1β can induce the expression of TF and von Willebrand factor on vascular endothelial cells [102]. In a mouse tumour model, TNF-α induced TF expression on endothelial cells. TNF-α and IL-1β have also been shown to downregulate and attenuate antithrombotic regulators such as thrombomodulin [103,104], a receptor on endothelial cells that binds with thrombin and activates protein C, a potent anticoagulant protein. The release of nitric oxide and prostacyclin—inhibitors of platelet adhesion and activation—from endothelial cells has also been shown to be suppressed after exposure to TNF-α and IL-1β [105,106]. Furthermore, TNF-α and IL-1β strongly impair the antithrombotic response of endothelial cells by stimulating the production of the fibrinolysis inhibitor, PAI-1.

Proangiogenic and growth factors such as vascular endothelial growth factor (VEGF), basic fibroblast growth factor, and granulocyte colony stimulating factor (G-CSF) also play a role in regulating the procoagulant phenotype of host cells. VEGF is secreted by various tumour cells and can induce TF expression on macrophages [107]. G-CSF has shown to lead to an increase in endothelial activation markers (thrombomodulin and von Willebrand factor) and coagulation markers, suggesting an increase in haemostatic activation [108]. Basic fibroblast growth factor has been shown to increase TF expression on endothelial cells [109].

#### 4.2.3. Adhesion Molecules

Cancer cells have the capacity to express specific adhesion molecules allowing for attachment to blood vessel walls and interactions with blood cells, as well as the activation of the procoagulant properties of host cells, primarily the endothelial cells, leukocytes, and platelets. The attachment of tumour cells to endothelial cells is significant in localising the initiation of clotting near the blood vessel wall and the subsequent formation of a thrombus. Several adhesion molecules have been described for the adhesion of different types of tumour cells to endothelial cells. For example, under flow conditions, HT-29M colon carcinoma cells used E-selectin to roll and adhere on activated endothelial cells. Vascular cell adhesion molecule-1 was required for the adhesion of A375M cells 29M [110]. These specific cancer and endothelial cell interactions can enhance aggregate formation which can promote clotting through perturbation of blood flow. Furthermore, endothelial cells and activated platelets express P-selectin to which cancer cells can bind; however, the ligand for P-selectin on cancer cells is unclear. In addition, cancer cells may facilitate interaction with platelets via three mechanisms [101]. These are through the integrin αV/β, with the platelet integrin αIIb/β3, or P-selectin on platelets binding to either glycoprotein s-Le(x) on mucin-producing carcinoma, or sulphatides, which are expressed on some cancer cells. The enhanced interactions between cancer cells and platelets and endothelial cells can promote cell–cell aggregate formation, leading to perturbation of blood flow, therefore promoting blood clotting and vessel occlusion.

#### 4.2.4. Neutrophil Extracellular Traps

The release of neutrophil extracellular traps (NETs) in response to pancreatic-cancer-derived factors has recently been reported in vitro [111]. NETs are a DNA-associated mesh of histones and neutrophil-derived proteases, which were first identified for their antimicrobial functions [112]. However, NETs have recently attracted interest for their ability to promote venous and arterial thrombosis in mice [113,114,115]. A significant reduction in venous thrombi was observed when NETs were targeted with DNAse I and neutrophil elastase inhibitor [113,114]. Cancer-associated NETs may further facilitate interactions with, or activation of, host cells to promote thromboembolic events. For example, NET-associated histones can activate endothelial cells and subsequently increase von Willebrand factor release (a glycoprotein important for platelet adhesion and aggregation in thrombosis) [114,116]. NETs can also serve as a platform for direct platelet adhesion and aggregation [6,117], which is vital for thrombus formation. A recent study by Mauracher et al. found that increased citrullinated histone H3 (a biomarker for NET formation) was associated with an increased incidence of VTE in patients with cancer, while other NET biomarkers (cell-free DNA and nucleosomes) were associated with high risk of VTE during the first 3–6 months [118]. These data suggest the significance of NETs in the pathogenesis of cancer-associated thrombosis.

#### 4.2.5. Mucins

Several types of cancers are a mucin-producing carcinoma with aberrant expression and altered glycosylation of several mucins [119,120]. The heavily O-linked glycosylation sites on mucins serve as ligands for selectins [121]. Mucins have been shown to interact with blood cells via selectins, resulting in the formation of microthrombi. Mucins alone, purified from xenografted tumours, were not capable of directly activating platelets in vitro; however, mucins incubated with whole blood caused platelet activation, an effect that was found to be mediated by leukocyte L-selectin [122]. In addition, purified mucins when injected intravenously into mice resulted in widespread platelet-rich intravascular microthrombi [122]. Shao and colleagues provided further mechanistic insight on mucin-induced microthrombi [123]. The reciprocal interaction between platelets and neutrophils, with L-selectin and P-selectin glycoprotein ligand-1 on neutrophils and P-selectin on platelets, was essential for the release of cathepsin G from neutrophils [123], a known agonist of platelet aggregation [124].

#### 4.2.6. Hypoxia

Tumours provide a highly hypoxic microenvironment [125,126], a condition that promotes endothelial dysfunction. In response to hypoxia, endothelial cells produce elevated levels of phospholipase A2, leading to the excessive production of prostaglandins and synthesis of platelet-activating factor (PAF) [127]. PAF is not only a potent platelet agonist but also activates neutrophils, promoting their adhesion to the endothelium under hypoxic conditions [128,129]. Indeed, blocking of PAF resulted in decreased neutrophil adhesion to hypoxic endothelial cells [129]. Moreover, hypoxia causes Weibel–Palade bodies present within endothelial cells to exocytose, resulting in the release of von Willebrand factor and overexpression of P-selectin, thereby increasing the indirect procoagulant response through endothelial cells. In addition, hypoxia can also increase ADP generated by tumours [130], further exacerbating platelet activation.

#### 4.2.7. Damage-Associated Molecular Patterns (DAMPs)

DAMPs are released by dying tumour cells or through cell stress pathways that do not necessarily lead to cell death. Their role has just started to become understood in cancer [131]. They represent a broad array of molecules such as histones, High Mobility Group Box 1 (HMGB1), S100 proteins, and heat shock proteins, which are localised within the cell and only released upon cell death or during cell stress. The release of DAMPs initiates a host response via innate immune pattern recognition receptors to coordinate protective responses. However, DAMPs may also have detrimental effects for the host, triggering chronic inflammation and immune cell activation [132], which may ultimately have consequences for thrombosis in addition to benefiting tumour growth and survival. Indeed, increased circulating nucleosomes are observed in plasma of cancer patients compared with in healthy controls. The DAMPs which have so far been recognised to have potent procoagulant activity are histones and HMGB1. The implications of circulating histone and HMGB1 in the blood circulation of cancer patients are increased platelet activation and aggregation [133,134] and activation of neutrophils, particularly the initiation of the release of NETs [135], which have been shown to exert major consequences for thrombosis as described above. Furthermore, higher extracellular DNA levels were observed in patients with PE which was specific for PE amongst other potential diagnoses [136], establishing a link between extracellular DNA and thromboembolic complications. However, these studies have not been conducted comparing patients with and without cancer.

#### 4.2.8. Cancer-Associated Chemotherapy

Despite a large body of evidence suggesting that cisplatin-based chemotherapy predisposes patients to thromboembolic events, the mechanism of cisplatin-associated thrombosis is not fully understood. Lechner et al. reported that cisplatin treatment of two human endothelial cell lines resulted in endothelial cell apoptosis, with the progressive release of procoagulant endothelial microparticles that generated thrombin independently of TF [137]. Chemotherapy is thought to increase the risk of VTE through direct drug-induced damage to the endothelium as well as by increasing the expression of TF procoagulant activity of monocytes and macrophages, which induces a procoagulant response from the host cells [35,138]. Another prothrombotic mechanism involving chemotherapy is its direct hepatotoxicity, which consequently leads to a decline in the plasma levels of natural anticoagulant proteins, including protein S, protein C, and antithrombin [35,139]. Chemotherapy is also responsible for inducing apoptosis of both tumour and host endothelial cells, causing cytokine release, which can increase both the expression and activity of TF (reviewed by Haddad and Greeno [45]).

#### 4.2.9. Coagulation Gene Defects

In a study observing thrombophilic genes in gastrointestinal cancers, Pihusch and colleagues found an increased presence of the factor V Leiden and prothrombin gene G20210A mutation in those who subsequently developed thromboembolic disease [140].

#### 4.2.10. Decreased Coagulation Inhibitors

An early study investigating the haemostatic balance in pancreatic cancer found that the coagulation inhibitors antithrombin III, heparin cofactor II, protein C, free protein S, and thrombomodulin were significantly decreased during the progression of pancreatic cancer after diagnosis [141].

## 5. Patient Management

Cancer patients have a significantly higher risk of developing VTE as compared to non-cancer patients. This is due to a combination of cancer-related, treatment-related, and patient-related factors. The pathophysiology of cancer-associated thrombosis is multifactorial and poorly understood. Patients with cancer-associated thrombosis have significantly shorter overall survival than cancer patients without thrombosis. All cancer patients should be periodically assessed for VTE risk. The risks of cancer-associated thrombosis increase with hospitalisation, infection, chemotherapy, blood transfusions, erythropoiesis-stimulating agents, the presence of medical comorbidities, and the presence of central venous catheters. Cancer patients undergoing major abdominal or pelvic surgery should be offered postoperative VTE prophylaxis for up to 4 weeks. Healthcare professionals should educate patients about the signs and symptoms of VTE. A high index of suspicion is required for the early diagnosis of cancer-associated thrombosis, and early initiation of treatment can improve survival.

## 6. Cancer-Associated Thrombosis Therapy

A detailed discussion concerning therapeutic management of cancer-associated thrombosis is beyond the scope of this review. The reader is referred to several exhaustive reviews on this topic, which cover the use and appropriateness of thromboprophylaxis in cancer patients undergoing surgery and in the ambulatory patient setting, as well as clinical strategies for acute and extended treatment (>6 months) and treatment issues within special populations (patients with recurrent VTE while on anticoagulation, patients with thrombocytopenia, patients with brain tumours, catheter-related thrombosis, and incidental cancer-related thrombosis) [142,143]. Low-molecular-weight heparin has been shown to be superior to warfarin in reducing the risk of current VTE in patients with cancer-associated thrombosis and is the recommended current standard thromboprophylactic treatment [142,143,144] and first-line therapy for acute cancer-associated thrombosis in several existing major clinical guidelines [145,146]. For VTE and cancer, the CHEST guideline and expert panel report [146] suggests usage of low-molecular-weight heparin over vitamin K antagonists (moderate-quality evidence, grade 2B), and low-molecular-weight heparin over direct-acting oral anticoagulants, dabigatran (Grade 2C), rivaroxaban (Grade 2C), apixaban (Grade 2C), or edoxaban (Grade 2C). Until recently there has been a paucity of evidence with respect to the use of direct-acting oral anticoagulants for treating cancer-associated thrombosis and whilst their use relative to low-molecular-weight heparin would have practical advantages, concerns remain around the lack of reversal agents to rapidly allow haemostasis and the lack of widely available assays to monitor their anticoagulant activity [142]. Recently, the first large randomised prospective clinical trial, the Hokusai VTE Cancer study, was published comparing the safety and efficacy of dalteparin (low-molecular-weight heparin) with edoxaban (a direct-acting Factor Xa inhibitor) [147]. Oral edoxaban was non-inferior to subcutaneous dalteparin with respect to a composite outcome of recurrent VTE or major bleeding. The rate of recurrent VTE was lower with edoxaban but with a higher rate of major bleeding. In a smaller clinical trial with a similar patient profile to Hokusai VTE Cancer study, Young et al. published the results of SELECT-D trial in which they compared oral Factor Xa inhibitor (rivaroxaban) with low-molecular-weight heparin in cancer patients with VTE. They concluded that rivaroxaban was associated with low VTE recurrence but higher clinically relevant nonmajor bleeding [131].

## 7. Conclusions

In recent years there have been significant advances in our understanding of the molecular mechanisms associated with the increased risk of VTE in cancer, although there remain significant gaps in our knowledge of the causes of and best approaches for thromboprophylaxis in cancer-associated thrombosis. More research in this field should lead to a better understanding of the pathophysiology and optimal therapeutic approaches for the prevention of cancer-related thrombosis.
